# Complete response of lung metastases from rectal cancer to combination first-line therapy of S-1 and irinotecan plus bevacizumab: A case report and review of the literature

**DOI:** 10.3892/ol.2014.1939

**Published:** 2014-03-05

**Authors:** JIRO SHIMAZAKI, GYO MOTOHASHI, KIYOTAKA NISHIDA, TAKANOBU TABUCHI, HIDEYUKI UBUKATA, TAKAFUMI TABUCHI

**Affiliations:** Department of Gastrointestinal Surgery, Ibaraki Medical Center, Tokyo Medical University, Ami, Ibaraki 300-0395, Japan

**Keywords:** metastatic colorectal cancer, chemotherapy, S-1

## Abstract

This report presents the case of a 72-year-old male who had undergone abdominoperineal resection following a diagnosis of lower rectal cancer with multiple lung metastases. Pathologically, the resected specimen exhibited advanced rectal cancer with regional lymphoid metastases and was classified as stage IV disease. S-1 and irinotecan (IRIS) plus bevacizumab combination therapy was used to treat the lung metastases following the surgery. S-1 (100 mg/body) was administered orally on days 1–14 of a 28-day cycle, and irinotecan (125 mg/m^2^) and bevacizumab (7.5 mg/kg) were administered by intravenous infusion on days 1 and 15. Computed tomography revealed a marked decrease in the size of the metastases following three therapeutic courses, and no lung metastases or new lesions were detected following nine therapeutic courses. The response was declared clinically complete. The patient refused additional treatment following nine therapeutic courses, and there was no recurrence 36 months after the final course of therapy. This case demonstrates the efficacy of IRIS plus bevacizumab as a first-line combination therapy against lung metastases of rectal cancer.

## Introduction

As a result of marked improvement in systemic chemotherapy against unresectable and/or recurrent colorectal cancer, the median survival time of patients with metastatic colorectal cancer has improved to >20 months with administration of fluorouracil (5-FU), irinotecan and oxaliplatin (1). According to the Japanese guidelines for the treatment of unresectable and/or recurrent colorectal cancer (2), 5-FU, leucovorin and oxaliplatin combination chemotherapy (FOLFOX), capecitabine and oxaliplatin combination chemotherapy (CapeOX), and 5-FU, leucovorin and irinotecan combination chemotherapy (FOLFIRI), are recommended as first-line chemotherapy regimens. Additionally, these regimens plus anti-vascular endothelial growth factor monoclonal antibody (bevacizumab) or anti-epidermal growth factor receptor monoclonal antibody have improved the median survival time of patients. At present, combination chemotherapy with the oral fluoropyrimidine S-1 has been shown to be effective against metastatic colorectal cancer (3–9). This report presents a case that demonstrates the efficacy of S-1 and irinotecan (IRIS) plus bevacizumab combination therapy against lung metastases of rectal cancer and discusses the relevant literature. Patient provided written informed consent.

## Case report

**Figure 1:**
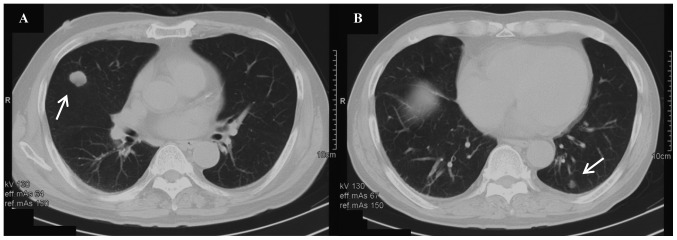
Chest computed tomography revealing two metastatic lung tumors. (A) A tumor measuring 25 mm in diameter is present in the middle lobe of the right lung (arrow); and (B) a tumor measuring 10 mm in diameter is observable in the lower lobe of the left lung (arrow).

**Figure 2:**
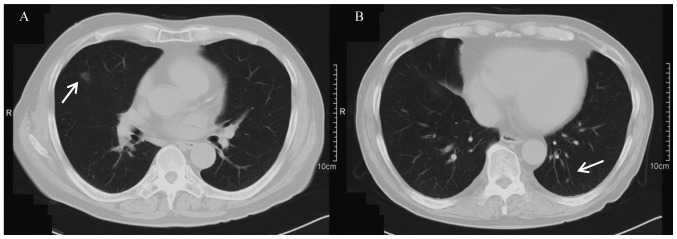
Chest computed tomography following three courses of therapy. (A) The tumor in the right lung has decreased in size to ~10 mm in diameter (arrow); and (B) the tumor in the left lung has decreased in size to ~3 mm in diameter (arrow).

**Figure 3:**
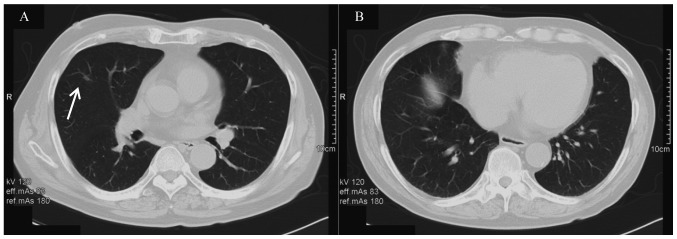
Chest computed tomography following six courses of therapy. (A) The tumor in the right lung has become scar tissue (arrow); and (B) no metastasis is observable in the left lung.

**Figure 4:**
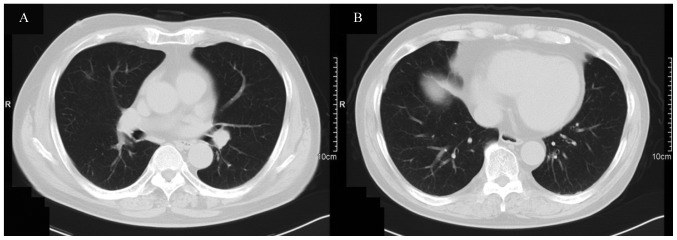
Chest computed tomography following nine courses of therapy. There is no metastatic tumor in the (A) right lung or (B) left lung.

A 72-year-old male visited a local physician with a 20-day history of progressive abdominal distension and bloody stool. The following day the patient was referred to Ibaraki Medical Center (Ami, Japan) with a diagnosis of rectal cancer. Patient medical history was otherwise unremarkable. On physical examination, all findings were unremarkable with the exception of slight pallor in the palpebral conjunctiva. Hematological investigations revealed anemia (hemoglobin levels, 9.6 g/dl; hematocrit, 28.4%). Other laboratory tests and serum levels of carcinoembryonic antigen and carbohydrate antigen 19-9 were all within normal limits. Colonoscopy revealed the entire circumference of an elevated tumor with central depression and erosion at the lower rectum. A biopsy specimen from the tumor was indicative of a moderately differentiated adenocarcinoma. Abdominal computed tomography (CT) revealed thickening of the rectal wall with regional lymph node swelling but no liver metastasis. Chest CT revealed two metastatic lung tumors measuring 25 mm (in the middle lobe of the right lung) and 10 mm in diameter (in the lower lobe of the left lung) (Fig. 1). A diagnosis of rectal cancer with multiple lung metastases was made, and abdominoperineal resection with lymph node dissection was performed in October 2009. Light microscopy revealed that the tumor had infiltrated the deep tissue layer through the muscularis propria layer of the rectum and that there were cancer metastases in 12 of the 14 lymph nodes. The tumor was diagnosed as stage IVA (T3, N2b, M1a) according to the International Union Against Cancer Tumor Node Metastasis classification (7th edition) (10). The patient refused resection of the lung metastases and placement of a peripherally inserted central venous (CV) port, and was hesitant to be treated with oxaliplatin and capecitabine due to potential peripheral neuropathy and hand-foot syndrome as side effects. On providing informed consent, the patient was administered IRIS plus bevacizumab combination therapy against the lung metastases. S-1 (100 mg/body) was administered orally on days 1–14 of a 28-day cycle, and irinotecan (125 mg/m^2^) and bevacizumab (7.5 mg/kg) were administered by intravenous infusion on days one and 15. Following three courses of therapy, the metastatic right lung tumor decreased in size to ~10 mm in diameter, and the left lung tumor had decreased in size to ~3 mm in diameter (Fig. 2). Following six courses of therapy, the metastatic right lung tumor had become scar tissue and no metastases could be detected in the left lung (Fig. 3). Following nine courses of therapy, no metastatic lung tumors could be identified (Fig. 4). The response was declared clinically complete. The patient refused additional treatment following nine courses of therapy, and there was no recurrence 36 months after the final course of therapy.

## Discussion

Multidisciplinary therapies, including systemic chemotherapy against unresectable and/or recurrent colorectal cancer, have improved, and FOLFOX, CapeOX and FOLFIRI combination chemotherapies plus bevacizumab or anti-EGFR monoclonal antibody can improve the survival times of patients. In the present case, the patient received IRIS plus bevacizumab combination therapy against lung metastases of rectal cancer. Regarding IRIS chemotherapy, Muro *et al* (3) reported that IRIS was not inferior to FOLFIRI in terms of progression-free survival when administered as second-line chemotherapy for patients with metastatic colorectal cancer. Colucci *et al* (4) and Tournigand *et al* (5) identified no significant differences in the median survival times of patients regardless of whether FOLFOX or FOLFIRI was selected as a first-line chemotherapy. As the noninferiority of IRIS with regard to FOLFIRI as a second-line chemotherapy has been proven, IRIS is considered to be a first-line chemotherapy for metastatic colorectal cancer. Futhermore, according to the European Society for Medical Oncology (11), IRIS is recommended as an additional therapeutic option for first-line chemotherapy in metastatic colorectal cancer.

The effectiveness of IRIS plus bevacizumab combination therapy as a first-line therapy for metastatic colorectal cancer has been reported. In a phase II study, Komatsu *et al* (12) reported that the response rate (complete response + partial response) was 57.7% and the disease control rate (complete response + partial response + stable disease) was 90.4%. Kato *et al* (13) reported that the response rate was 62.0 versus 72% and progression-free survival time was 324 versus 345 days for FOLFIRI plus bevacizumab versus IRIS plus bevacizumab, respectively. In terms of side effects and safety, Yamada *et al* (14) reported that the IRIS plus bevacizumab regimen was tolerated: Grade 3/4 neutropenia was observed in 26% patients, grade 3/4 anorexia was observed in 12% and grade 3/4 diarrhea was observed in 8%. In the present case, the efficacy of IRIS plus bevacizumab combination therapy was confirmed by the decrease in size of the metastatic right and left lung tumors from ~25 mm to 10 mm and ~10 mm to 3 mm in diameter, respectively, following three courses of therapy. The tumor regression rate was ~62.8%, and the right lung tumor became scar tissue following six courses of therapy. There were no metastatic tumors in the lungs following nine courses of therapy. The patient exhibited grade 1 depilation, but there were no problems with tolerability.

Treatment of lung metastases from colorectal cancer is a debated issue. The overall survival of patients with completely resectable lung metastases is better than that of patients with unresectable lung metastases (15–17). The Japanese guidelines for treatment of lung metastases from colorectal cancer recommend surgery if the primary lesion and lung metastases are completely resectable and the performance status of the patient suggests surgery may be tolerated (2). According to the Japanese guidelines, resection was recommended for the lung metastases in the present case; there were two lung metastases and the performance status of the patient was good. However, the patient selected chemotherapy against the lung metastases, refusing pneumonectomy and the adjuvant chemotherapy that would be required following surgery if the pneumonectomy was performed. The patient selected IRIS plus bevacizumab combination first-line therapy for the following reasons: i) High antitumor effectiveness has been reported in a previous study (although it was a phase II study) (13); ii) a CV port is not necessary; and iii) unlike 5-FU therapy, patients may be released from the infusion pump while at home. With regard to the lack of requirement for a CV port and infusion pump, CapeOX plus bevacizumab therapy was an option for first-line therapy in the present case. However, hand-foot syndrome as a side effect of capecitabine and peripheral neuropathy as a side effect of oxaliplatin are frequently observed, and management of these side effects is essential (18,19). In the present case, the patient was hesitant to be treated with oxaliplatin and capecitabine due to these side effects. Therefore, better tolerance of IRIS plus bevacizumab combination therapy than of CapeOX plus bevacizumab combination therapy can be expected in cases of metastatic colorectal cancer.

In conclusion, IRIS plus bevacizumab combination therapy is well tolerated and efficacious as first-line chemotherapy for metastatic colorectal cancer. As a CV port is not required and patients can be released from the infusion pump while at home, IRIS plus bevacizumab combination therapy could contribute to improved quality of life in patients with metastatic colorectal cancer.
